# On Outage Probability and Ergodic Rate of Downlink Multi-User Relay Systems with Combination of NOMA, SWIPT, and Beamforming

**DOI:** 10.3390/s20174737

**Published:** 2020-08-21

**Authors:** Ta Chi Hieu, Nguyen Le Cuong, Tran Manh Hoang, Do Thanh Quan, Pham Thanh Hiep

**Affiliations:** 1 Faculty of Radio-Electronic Engineering, Le Quy Don Technical University, 236 Hoang Quoc Viet, Hanoi 100000, Vietnam; hieunda@mta.edu.vn (T.C.H.); dtquan82@gmail.com (D.T.Q.); 2 Faculty of Electronics and Telecommunications, Electric Power University, 238 Hoang Quoc Viet, Hanoi 100000, Vietnam; cuongnl@epu.edu.vn; 3 Faculty of Profesional Telecommunications, Nha trang University of Telecommunication, 101 Mai Xuan Thuong, Nha Trang 650000, Vietnam; hoangsqtt@gmail.com

**Keywords:** non-orthogonal multiple-access, energy harvesting, radio frequency, successive-interference cancellation, power-beacon

## Abstract

A downlink of multi-user non-orthogonal multiple access (NOMA) relay systems is considered. To improve system performance, relay nodes are used to forward signals from the base station (BS) to the end users, and they are wirelessly powered by energy harvesting from the radio frequency transmitted from the BS. Moreover, beamforming is applied at the BS based on multiple antennas and relay nodes consist of one transmit antenna but several receive ones. The system performance is demonstrated through closed-form expressions of outage probability (OP) and ergodic rate (ER) over Rayleigh fading channels. The proposed system is investigated in two cases of perfect and imperfect successive interference cancellation (SIC), and the imperfect channel state information condition is also taken into consideration. The OP and ER are calculated in many scenarios and the optimal time fraction of energy harvesting corresponding to the minimum OP is discussed. The exact and approximate theoretical results are compared with the simulation result to confirm the proposed theoretical analysis method.

## 1. Introduction

The Internet of Things (IoT) has received an increasing attention from both industry and academia for recent years [1,2,3]. It is considered to be a crucial means for wireless connections in the era of the fourth industry. The IoT was also included in the advanced wireless standards such as the fourth generation (4G) and has been considering for the fifth generation (5G) of mobile communications [4,5]. Most IoT devices are battery operated or can be located in the deep ocean, outback, and military devices, therefore, battery recharging methods can be economically infeasible or unredeemable. As a result, the IoT system needs to be improved its performance such as lifetime, capacity, spectral and energy efficiencies to meet with requirements of future communication systems.

To support a large multi-user system such as the IoT, the non-orthogonal multiple access (NOMA) is significantly considered due to its high bandwidth efficiency [6,7,8]. These works investigated the performance of the multi-user NOMA system and solved problems through simulation. The coexistence of both OMA and NOMA methods for communication capability on a communication device in wireless networks is proposed in [9], i.e., according to requirement of Quality of Service (QoS), users decide to use OMA or NOMA method for information transmission. This scheme would be risky because of inaccurate recognition of QoS. On the other hand, the problems of mode selection, dynamic user association, and power optimization have been studied for in-band full duplex (IBFD) and NOMA operating networks [10].

There has been a research that takes multi-beam multiple input single output (MISO) NOMA into consideration [11]. However, the author assumed that each cluster has only two users named as the near user and far user. We are going to extend this work with more users in the same beam.

A combination of the NOMA and energy harvesting (EH) was considered in some papers [12,13,14,15,16]. In these papers, the authors proposed the simultaneous wireless information and power transfer (SWIPT) for the NOMA network in which the near user works as the EH relay to assist the far user for improving both network lifetime and spectral efficiency.

Moreover, there have been several papers researching on a combination of the radio frequency (RF) EH and relay communication to enhance the system performance [17,18,19,20,21]. For example, in [17], the dual-hop decode-and-forward (DF) relaying network employing a time switching-based relaying mechanism was analyzed and the proposed system was evaluated via the outage probability. In [18], the authors concentrated on the amplify-and-forward (AF) relaying system and investigated the fixed-gain and variable-gain relaying schemes to deal with interference. In [19], the multiple input multiple output (MIMO) full-duplex system was considered and the optimal throughput was analyzed. Since antennas are configured at relay nodes, the energy consumption of relay nodes depends on the number of antennas.

The small cell station can harvest energy transmitted by a macro-cell base station or by solar panels [22]. This work is close to our proposed system; however, it only investigated EH for the small cell station of a two-tier heterogeneous small cell network, and the authors did not take the NOMA aspect into consideration. On the other hand, to harvest energy from RF signal or solar panels, the base station uses different architectures, it is hard to combine in one device. The problem of EH efficiency from RF is shown in [23], in this work, the authors proposed a low complexity energy transfer and transmission adaptation algorithm in order to determine the optimal power station’s transmission power. However, this work considered an orthogonal frequency multiple access scheme.

A combining SWIPT-NOMA downlink relaying protocol is a potential approach to achieve high energy efficiency and performance, i.e., prolonging the lifetime and improving the throughput of wireless networks, especially for designing the future 5G and 6G networks. To the best of our knowledge, there is a lack of studies on a combination of the SWIPT and NOMA in relaying network systems.

Due to a big gap between the active sensitivity of energy harvester and that of decoder (i.e., −10 dBm for EH and −60 dBm for information receiving), the SWIPT-based network is only appropriate for short-distance transmission. To overcome this limitation, the authors of [24,25,26] proposed the systems in which individual EH and information receivers are used depending on the time switching (TS) and power slitting (PS) to supply power for wireless devices. In particular, [27] proposed three wireless power transfer policies. Namely, the cooperative power beacons (PBs) power transfer, the best PBs power transfer, and the nearest PBs power transfer. Since the SWIPT with PBs is fully controllable, it can provide various applications with high quality of service (QoS) requirements. The RF EH from PBs was proposed and investigated in [28,29]. However, [28,29], and also other previous works [25,26] only considered point-to-point communication systems and frequencies used to transmit PBs and information during EH period are different. In these papers, the PBs are transmitted continuously; however, this approach is not an optimal solution in the sense of saving the PBs power and requires a high practical cost as well.

It is clear that an application of the EH from the surrounding RF to supply power for wireless devices is an avocational research for the next generation networks, 5G and 6G. In our initial works [30,31], we have investigated the NOMA beamforming relay system and then concentrated on a combination of the SWIPT, NOMA and beamforming technique for the downlink of the multi-user system at the beginning step.

The contributions of this paper are summarized as follows

- We propose a downlink MIMO-NOMA with SWIPT relay system which aims to improve the energy and spectrum efficiencies of the system. To reduce the complexity and mitigate inter-user interference, users in the system are divided into several clusters and a suitable user clustering mode is found out. The relays are employed to mitigate the effect of fading and reduce the number of training sequences for estimating the CSI at the base station.
- We derive the closed-form expressions of the outage probability (OP) and ergodic rate (ER) for each user in any cluster. For the practical purpose, we investigate the proposed MIMO-NOMA with SWIPT relay system over Rayleigh channel. The time duration of EH is optimized in the sense of minimum OP. The analysis result provides insights to understand the performance of the MIMO-NOMA system with SWIPT through the mathematical expressions.
- We consider the system in the case of imperfect channel state information (CSI) caused by the downlink channel estimation error and both cases of perfect and imperfect successive interference cancellation (SIC). The result shows that the system performance is reduced significantly due to the imperfect CSI and imperfect SIC. All analysis results are compared with simulation results to confirm the correctness of the derived mathematical expressions.

The rest of the paper is organized as follows. Section 2 represents the proposed system model, illustrating CSI requirement, channel model, and energy harvesting technique. Section 3 concentrates on performance analysis, deriving the closed-form expressions of the OP and ER. Section 4 demonstrates numerical results and discussion. Finally, Section 5 concludes the paper.

For the convenience, in the following Table 1, we provide notations along with their descriptions used in this paper.

## 2. System Model

**Figure 1:**
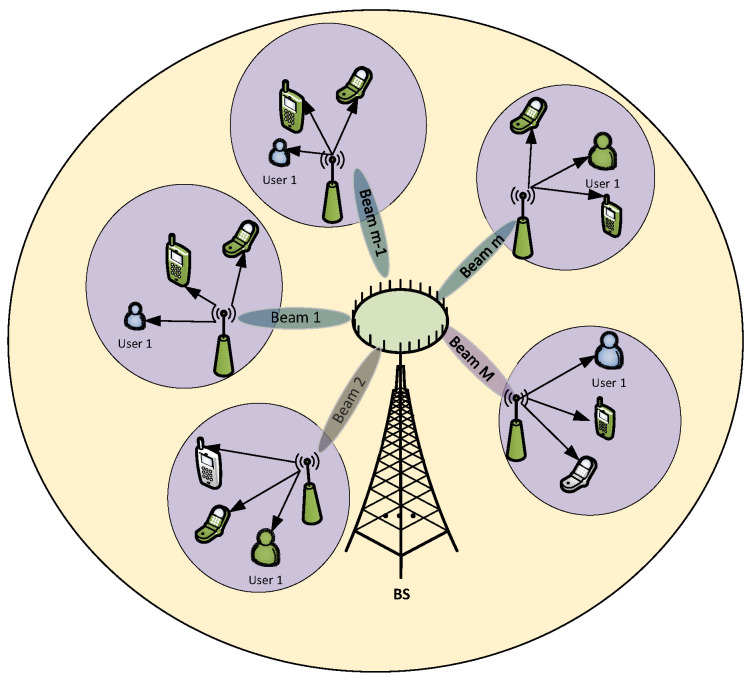
The proposed system model.

**Figure 2:**
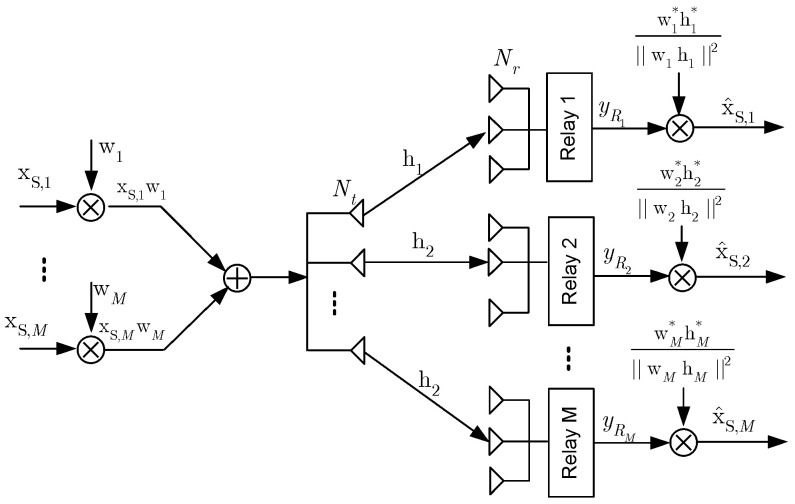
The block diagram of the first hop of the proposed multi-relay beamforming scheme.

In this paper, a downlink of the multi-user (MUs) relaying communication network is investigated. In this system, the BS communicates with users via a support of relay (R) nodes as illustrated in Figure 1. There are *M* relay nodes that are deployed in the cell and have constrained energy. To improve the energy efficiency, the relay node harvests energy from surrounding RF. The BS node and each relay node have $N_{t}$ and $N_{r}$ antennas, respectively, satisfying the following condition $\left(N_{t}\geq \left(M-1\right)\right)$. While users have a single antenna because of the limited size, the BS can be considered to be having massive antennas. The block diagram of the first hop from the BS node to the relay node is shown in Figure 2, in which the signal, which is transmitted to all users in the *m*th cluster, is denoted as $x_{S,m}$. The signal $x_{S,m}$ is multiplied by $N_{t}\times 1$ beamforming vector $w_{m}$ before being transmitted over channel, and the beamforming vector $w_{m}$ is assumed to be normalized $∥w_{m}{∥}^{2}=1$.

Users that are close to a relay node, are grouped into a cluster with the help of the GPS algorithm (The spatial direction of users can be found via various methods/technologies such as GPS or user location tracking algorithms.). We assume that there are *N* users in every cluster and the NOMA scheme is applied to improve the spectrum efficiency, i.e., signals are superposed in the power domain at the BS. Moreover, the NOMA techniques and DF protocol are performed at the relay node and users to remove the inter-user interference. In the next section, the proposed multi-relay NOMA scheme is described in detail.

The operation of the MIMO-NOMA system with SWIPT and beamforming in DF protocol can be summarized as follows. A complete signal transmission procedure from the BS to the $D_{n}$ under assistance of SWIPT relays in the proposed MIMO-NOMA system takes three time slots. In the first time slot, the relay harvests energy from broadcasts of BS over downlink channel. The duration time of this slot is denoted by αT. In the second time slot, the BS sends the signal to relays after performing superposition coding based on the characteristic of each user. In the third time slot, $R_{m}$ re-encodes the received signals and then assigns the power for these signals before forwarding them to the users.

### 2.1. CSI Condition and Channel Model

For transmitting beamforming, the BS requires the CSI. In the proposed model, the BS receives the CSI by up-link channel estimation. Herein, at the beginning of a working section, relay nodes send pilot symbols to the BS. Since the BS and relay node are imperfectly synchronized, all pilot signals transmitted from the relay node to the BS are non-orthogonal and imperfect. As a result, the perfect CSI obtained at the BS is absolutely challenging owing to both causes as channel estimation error and feedback delay. Assuming that the minimum mean squared error (MMSE) estimation is used, the difference between the estimated channel matrix ${\hat{h}}_{m}$ and the actual channel matrix $h_{m}$ can be illustrated as the following equation [33]

(1) $$\begin{array}{c} h_{m}={\hat{h}}_{m}+e, \end{array}$$

here e denotes the channel estimation error vector with independent and identically distributed (i.i.d.) zero mean and unit variance complex Gaussian distributed entries.

To allocate power for users, the relay node also require CSI. The difference between the estimated channel coefficient ${\hat{g}}_{n}$ and actual channel coefficient $g_{n}$ can be also demonstrated as

(2) $$\begin{array}{c} g_{n}={\hat{g}}_{n}+e, \end{array}$$

here *e* denotes an error of the channel coefficient estimation between the $R_{m}$ and the $D_{n}$.

In addition, ${\hat{\Omega }}_{A}=\Omega_{A}-\sigma_{\varepsilon_{A}}^{2}$ with $A\in \{{\mathrm{SR}}_{m},{\mathrm{RD}}_{n}\}$ is the channel gain norm of the ${\hat{h}}_{m}$. Let $0\leq \rho_{A}\leq 1$ is the correlation between coefficients of channel estimation error and coefficients of the perfect channel. Let $\sigma_{\varepsilon_{A}}^{2}=\rho_{A}\Omega_{A}$ and ${\hat{\Omega }}_{A}=\left(1-\rho_{A}\right)\Omega_{A}$ denote the variance norm and the variable of channel estimation error, respectively.

In this paper, the zero-force beamforming (ZFBF) is employed at the BS to deal with a trade-off between the complexity of implementation and performance of the system. The weight $w_{m}$ for the *m*th cluster is designed to cancel the interference from the other clusters.

Every antenna of the BS sends the superposition code, which consists of *N* signals of the *m*th cluster and can be described by $x_{m}$.

(3) $$\begin{array}{c} x_{m}=\sum_{n=1}^{N}\sqrt{a_{n}P_{S}}x_{m,n}, \end{array}$$

here $a_{n}$ illustrates the power allocation coefficient of the (m,n)th user with $\sum_{n=1}^{N}a_{n}=1$. At the output of antenna, this signal is built with the beam vector, $w_{m}$, that depends on the ZFBF. As a result, the signal corresponding to the *m*th cluster is given by the following equation.

(4) $$\begin{array}{c} x_{S,m}=w_{m}x_{S,m}=w_{m}\sum_{n=1}^{N}\sqrt{a_{n}P_{S}}x_{m,n}. \end{array}$$

The $N_{t}\times 1$ signal vector $x_{S}=x_{S,1}+\cdots +x_{S,M}$ is transmitted to all relay nodes over a single cell. Hence, the received signal vector at the relay node can be calculated as

(5) $$\begin{array}{cc} y_{R} & =h_{m}x_{S}+n_{m}\\ & =\underset{m\mathrm{th}\;\mathrm{beam}}{\underset{︸}{h_{m}w_{m}\sum_{n=1}^{N}\sqrt{a_{n}P_{S}}x_{m,n}}}+\underset{\mathrm{other}\;\mathrm{beam}}{\underset{︸}{h_{m}\sum_{k\neq m}x_{s,k}}}+n_{m}, \end{array}$$

where $n_{m}=\left[n_{1},\cdots ,n_{N}\right]\in C^{1\times N}$ with $n_{m}∼\mathrm{CN}\left(0,\sigma_{m}^{2}\right)$ denotes the additive white Gaussian noise (AWGN) at the *m*th relay (The channel gain formulation, the interference model, the signaling overhead can be found in [34]).

According to the NOMA technique, the power domain is used for multiple access of the MUs at the same time and frequency, i.e., different power levels are allocated to different users. Consequently, $w_{m}$ can be considered to be a projection of $h_{m}$ in a null space of interfered channels according to the *m*th cluster. To maximize the channel gain and mitigate the inter-cluster interference, the $w_{m}$ can be defined as

(6) $$\begin{array}{c} w_{m}=\frac{\Pi_{m}h_{m}}{∥\Pi_{m}h_{m}∥}, \end{array}$$

here $\Pi_{m}=I_{N}-H_{m}{\left(H_{m}^{H}H_{m}\right)}^{-1}H_{m}^{H}$, $H_{m}$ denotes an extended channel matrix that does not consist of $h_{m}$ and its construction is represented as

(7) $$\begin{array}{c} H_{m}={\left[h_{1},h_{2},\cdots ,h_{m-1},h_{m+1},\cdots ,h_{M}\right]}^{T}. \end{array}$$

The condition to transmit the signal from the BS to the *m*th relay node is that there is a dimension greater than zero in the null space of $H_{m}$. As a result, we have

(8) $$\begin{array}{c} h_{m}^{H}w_{j}=0,\forall m\neq j. \end{array}$$

In other words, the condition in (8) is used to cancel completely interference during of the first hop of inter-relay. In addition, owning to the assistance the multiple relays, the size of matrix for designing beamforming is not so large and the computational complexity is bearable.

In this paper, a beam is assumed to be designed perfectly. Therefore, the output signal of the SIC structure at the *m*th relay node can be illustrated as

(9) $$\begin{array}{cc} y_{R,SIC} & =h_{m}w_{m}\sum_{n=1}^{N}\sqrt{a_{n}P_{S}}x_{m,n}+n_{m}\\ & =\underset{\mathrm{desired}\;\mathrm{signal}\;\mathrm{of}\;\left(m,n\right)\mathrm{th}\;\mathrm{user}}{\underset{︸}{h_{m}w_{m}\sqrt{a_{n}P_{S}}x_{m,n}}}+\underset{\mathrm{interference}\;\mathrm{of}\;\mathrm{other}\;\mathrm{users}}{\underset{︸}{h_{m}w_{m}\sum_{i=n+1}^{N}\sqrt{a_{i}P_{S}}x_{m,i}}}+\underset{\mathrm{interference}\;\mathrm{of}\;\mathrm{imperfect}\;\mathrm{SIC}}{\underset{︸}{h_{m}w_{m}\sum_{k=1}^{n-1}\sqrt{\xi_{1}a_{k}P_{S}}x_{m,k}}}+n_{m}. \end{array}$$

The term $h_{m}w_{m}\sum_{k=1}^{n-1}\sqrt{\xi_{1}a_{k}P_{S}}x_{m,k}$ is equal to zero if the SIC is perfect.

### 2.2. Energy Harvesting

**Figure 3:**
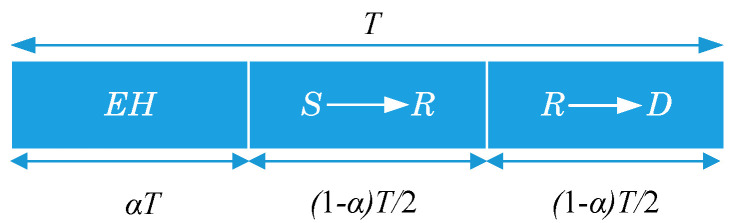
The time switching-based relaying (TSR) protocol.

In this work, the time switching (TS) is investigated owning to its higher performance and easier implementation than the power splitting when these two protocols are applied for the multi-user MIMO networks with the EH technique as demonstrated in [35]. The TS architecture [36] as in Figure 3 is supposed for the operation of the relay node (The proposed analysis approach can also be applied to the power spitting EH model [37]). In this figure, *T* is a block time to transmit a certain block of information from the source node to the destination node. The communication process is split into three time slots, the first slot, αT (0≤α≤1), for energy harvesting at the relay node, the second one, (1−α)T/2, for the S-to-R information transmission and the third one, (1−α)T/2, for the R-to-D information transmission. In case the relay node does not have an energy buffer to store the harvested energy, the harvest-use (HU) architecture [38] can be used.

Assuming that the EH process is only performed from the received signals in αT time interval of each period. As a result, the harvested energy can be calculated as ([36], Equation (2)).

(10) $$\begin{array}{c} E_{h}=\alpha T\eta P_{S}{\left|h_{m}w_{m}\right|}^{2}, \end{array}$$

where η, 0<η<1, illustrates the energy conversion efficiency and depends on the harvester quality and $P_{S}$ denotes the transmission power of the source.

According to the HU architecture, the relay node uses total harvested energy in the harvesting phase to forward the received data to users. Hence, the transmission power of the relay node is given by

(11) $$\begin{array}{cc} P_{R} & =\frac{2\alpha \eta P_{S}}{\left(1-\alpha \right)}|h_{m}w_{m}{|}^{2}=\phi P_{S}{\left|h_{m}w_{m}\right|}^{2}, \end{array}$$

here ϕ=2αη/(1−α).

The large-scale fading coefficient of the (m,n)th user is denoted by $\sqrt{d_{n}^{-\beta }}$, where $d_{n}$ is the distance between the relay node and the (m,n)th user and β is the path loss factor. Moreover, ${\tilde{g}}_{n}$ represents the small-scale fading coefficient of the (m,n)th user, leading to the fact that $g_{n}={\tilde{g}}_{n}\sqrt{d_{n}^{-\beta }}$ and $g_{n}∼CN\left(\mu ,\Omega_{{\mathrm{RD}}_{n}}\right)$, where $\Omega_{{\mathrm{RD}}_{n}}=E\{|g_{n}{|}^{2}\}$. The AWGN noise vector at the (m,n)th user is $w_{D_{n}}∼CN\left(0,N_{0}\right)$ with $N_{0}$ is the noise variance at the receiver. All channel gains have the i.i.d Rayleigh distribution. Without any loss of generality, we assume that $d_{1}>d_{2},\ldots ,>d_{N}$. Hence, the channel gains are sorted according to the ascending order $|g_{1}{|}^{2}<,\ldots ,<{\left|g_{N}\right|}^{2}$.

During the second time slot, the relay node re-encodes and forwards the messages to the user. Since the transmission power of the relay node, which depends on the quality of the energy harvester, is always smaller than that of the source. Therefore, it is necessary to re-allocate power to be fair performance of users. The output signal of the SIC architecture of the (m,n)th user is given as

(12) $$\begin{array}{cc} y_{D_{n}}= & \underset{\mathrm{desired}\;\mathrm{signal}\;\mathrm{of}\;\left(m,n\right)\mathrm{th}\;\mathrm{user}}{\underset{︸}{g_{n}\sqrt{b_{n}P_{R}}x_{m,n}}}+\underset{\mathrm{interference}\;\mathrm{of}\;\mathrm{other}\;\mathrm{users}}{\underset{︸}{g_{n}\sum_{i=n+1}^{N}\sqrt{b_{i}P_{R}}x_{m,i}}}+\underset{\mathrm{interference}\;\mathrm{of}\;\mathrm{imperfect}\;\mathrm{SIC}}{\underset{︸}{\sum_{k=1}^{n-1}\sqrt{\xi_{2}b_{k}P_{R}}g_{n}x_{m,k}}}+w_{D_{n}}. \end{array}$$

At the relay node, the SIC removes the interference signal of the other users having stronger power, therefore, in the case of perfect SIC, the signal to interference plus noise ratio (SINR) of the (m,n)th user at the relay node is expressed by $\gamma_{R,n}$, and from (9), it can be calculated as

(13) $$\begin{array}{c} \gamma_{R,n}=\frac{P_{S}a_{n}|h_{m}w_{m}{|}^{2}}{\sum_{i=n+1}^{N}P_{S}a_{i}|h_{m}w_{m}{|}^{2}+\sigma_{R,n}^{2}}. \end{array}$$

In the case of imperfect SIC, due to channel estimation error and remaining interference, the term $h_{m}w_{m}\sum_{k=1}^{n-1}\sqrt{\xi_{1}a_{k}P_{S}}x_{m,k}$ in (9) is not equals to zero and it depends on the quality of the SIC structure. As a result, the instantaneous SINR in this case is given by

(14) $$\begin{array}{c} \gamma_{R,n}^{ipSIC}=\frac{P_{S}a_{n}|h_{m}w_{m}{|}^{2}}{\sum_{i=n+1}^{N}P_{S}a_{i}|h_{m}w_{m}{|}^{2}+{\left|h_{m}w_{m}\right|}^{2}\xi_{1}\sum_{k=1}^{n-1}a_{k}P_{S}+\sigma_{R}^{2}}, \end{array}$$

where $\xi_{1}$ represents the impact level of residual interference at the relay.

From (12), the SINR at the (m,n)th user in the case of perfect SIC is calculated as follows. Note that, in case the power for (m,i)th user is larger than that for (m,n)th user, i.e., n≥i or $b_{n}\leq b_{i}$, then the SINR of $x_{m,i}$ at the (m,n)th user can also be expressed as

γD,i={(15a)PRbi|gn|2∑k=i+1NbkPR|gn|2+σD,n2,if i < N,(15b)PRbi|gn|2σD,n2,if i = N.

When $x_{m,i}$ is decoded successfully, i.e., $\gamma_{D,i}\geq \gamma_{{\mathrm{th}}_{i}}$, the $x_{m,i}$ is removed from the (m,n)th user’s SIC structure, here $\gamma_{{\mathrm{th}}_{i}}$ is the threshold of SINR of the user. The SIC performs continuously at the (m,n)th user until its own signal is decoded successfully.

In the case of imperfect SIC, the SINR of $x_{m,i}$ at the (m,n)th user with n≥i is given as

(16) $$\begin{array}{c} \gamma_{D,i}^{ipSIC}=\frac{P_{R}b_{i}{\left|g_{n}\right|}^{2}}{\sum_{k=i+1}^{N}b_{k}P_{R}|g_{n}{|}^{2}+\sum_{j=1}^{i-1}\xi_{2}b_{j}P_{R}{\left|g_{n}\right|}^{2}+\sigma_{D,n}^{2}}. \end{array}$$

where $\xi_{2}$ represents the impact level of residual interference at the (m,n)th user.

Moreover, with the DF protocol, the end-to-end SINR is defined as the minimum value of the two hops, BS→R and $R\to D_{n}$, consequently, we have

(17) $$\begin{array}{c} \gamma_{e2e}=\min\left(\gamma_{R,n},\gamma_{D,n}\right). \end{array}$$

## 3. Performance Analysis

### 3.1. Outage Probability

The outage probability (OP) is defined as the probability of an event in which the transmission rate of the system is lower than the minimum required data rate. In this section, the OP of the considered (m,n)th user is derived in two cases of the perfect and imperfect SICs.

Let $r_{1}$ and $r_{n}$ (bit/s/Hz) denote the minimum required data rate of two links, BS→R and $R\to D_{n}$, respectively. To simplify calculations, set $r_{1}=r_{n}=r$. Therefore, the OP of the (m,n)th user can be defined as

(18) $$\begin{array}{cc} {\mathrm{OP}}_{D_{n}}= & \mathrm{Pr}\left[\frac{1-\alpha }{2}\log_{2}\left(1+\gamma_{e2e}\right)\right]\\ = & \mathrm{Pr}\left(\gamma_{e2e}<2^{\frac{2r}{1-\alpha }}-1\right) \end{array}$$

By substituting $\gamma_{e2e}$ from (17) into (18), the OP of (m,n)th user can be calculated as

(19) $$\begin{array}{cc} {\mathrm{OP}}_{D_{n}} & =\mathrm{Pr}\left[\min\left(\gamma_{R_{n}},\gamma_{D_{n}}\right)<\gamma_{\mathrm{th}}\right]\\ & =1-\mathrm{Pr}\left(\gamma_{R_{n}}\geq \gamma_{\mathrm{th}},\gamma_{D_{n}}\geq \gamma_{\mathrm{th}}\right), \end{array}$$

here $\gamma_{\mathrm{th}}=2^{\frac{2r}{1-\alpha }}-1$.

Based on the equations of SNR given in (13) and (15a), the OP in case of perfect SIC can be calculated as in (20).

(20) $$\begin{array}{c} {\mathrm{OP}}_{D_{n}}=1-\mathrm{Pr}\left(\frac{P_{S}a_{n}|h_{m}w_{m}{|}^{2}}{\sum_{i=n+1}^{N}P_{S}a_{i}|h_{m}w_{m}{|}^{2}+\sigma_{R}^{2}}\geq \gamma_{\mathrm{th}},\frac{P_{R}b_{n}{\left|g_{n}\right|}^{2}}{\sum_{i=n+1}^{N}b_{i}P_{R}{\left|g_{n}\right|}^{2}+\sigma_{D,n}^{2}}\geq \gamma_{\mathrm{th}}\right), \end{array}$$

In the case of imperfect SIC, from (14) and (16), the outage probability is derived as follows.

(21) $$\begin{array}{c} {\mathrm{OP}}_{D_{n}}^{ipSIC}=1-\mathrm{Pr}\left(\begin{array}{c} \frac{P_{S}a_{n}|h_{m}w_{m}{|}^{2}}{\sum_{i=n+1}^{N}P_{S}a_{i}|h_{m}w_{m}{|}^{2}+{\left|h_{m}w_{m}\right|}^{2}\xi_{1}\sum_{k=1}^{n-1}a_{k}P_{S}+\sigma_{R}^{2}}\geq \gamma_{\mathrm{th}},\\ \frac{P_{R}b_{n}{\left|g_{n}\right|}^{2}}{\sum_{i=n+1}^{N}b_{i}P_{R}|g_{n}{|}^{2}+\sum_{k=1}^{n-1}\xi_{2}b_{k}P_{R}{\left|g_{n}\right|}^{2}+\sigma_{D,n}^{2}}\geq \gamma_{\mathrm{th}} \end{array}\right). \end{array}$$

Set $X=|h_{m}w_{m}{|}^{2}$ and $Y=|g_{n}{|}^{2}$ for notation convenience. Since the normalized $w_{m}$ is designed independently with $h_{m}$, the $|h_{m}w_{m}{|}^{2}$ is Chi-square distributed with 2K degree of freedom (If a variable is random and has Chi-square distribution with *K* degree of freedom, it becomes sum of Rayleigh distribution ([39], p. 16)), and $K=N_{t}\left(N_{r}-\left(M+1\right)\right)$[33], and $|g_{n}{|}^{2}$ is Chi-square distributed with two degree of freedom. Hence, the CDF and PDF of *X* are given as

(22) $$\begin{array}{c} F_{X}\left(x\right)=1-\exp\left(-\frac{x}{\left(1-\rho \right)\Omega_{\mathrm{SR}}}\right)\sum_{k=0}^{K-1}\frac{1}{k!}{\left(\frac{x}{\left(1-\rho \right)\Omega_{\mathrm{SR}}}\right)}^{k}, \end{array}$$

(23) $$\begin{array}{c} f_{X}\left(x\right)=\frac{x^{K-1}}{\Gamma \left(K\right){\left[\left(1-\rho \right)\Omega_{\mathrm{SR}}\right]}^{K}}\exp\left(-\frac{x}{\left(1-\rho \right)\Omega_{\mathrm{SR}}}\right). \end{array}$$

Based on the ordered statistics, the PDF of channel gain $|g_{n}{|}^{2}$ can be written as ([40], Equation (6.58)).

(24) f|gn|2(y)=N!(N−n)!(n−1)!∑j=0N−n(−1)jN−njf|gj|2(y)F|gj|2(y)n+j−1,

where

(25) $$\begin{array}{c} f_{|g_{j}{|}^{2}}\left(y\right)=\frac{1}{\left(1-\rho \right)\Omega_{{\mathrm{RD}}_{j}}}\exp\left(-\frac{y}{\left(1-\rho \right)\Omega_{{\mathrm{RD}}_{j}}}\right), \end{array}$$

(26) $$\begin{array}{c} F_{|g_{j}{|}^{2}}\left(y\right)=1-\exp\left(-\frac{y}{\left(1-\rho \right)\Omega_{{\mathrm{RD}}_{j}}}\right). \end{array}$$

The corresponding CDF of $|g_{n}{|}^{2}$ is given by

(27) F|gn|2(y)=N!(N−n)!(n−1)!∑j=0N−n(−1)jn+jN−nj1−exp−y(1−ρ)ΩRDjn+j,

Using the binomial expansion for (27) we have

(28) F|gn|2(y)=N!(N−n)!(n−1)!∑j=0N−n(−1)jn+jN−nj∑ℓ=0n+j(−1)ℓn+jℓexp−ℓy(1−ρ)ΩRDj.

As a result, the OP in two cases of the perfect and imperfect SICs is evaluated through the CDF and PDF of both S-R and R-D hops.

The Equation (20) can be represented as

(29) $$\begin{array}{c} {\mathrm{OP}}_{D_{n}}\overset{\Delta }{=}1-\mathrm{Pr}\left(X\geq \frac{\gamma_{\mathrm{th}}\sigma_{R}^{2}}{P_{S}\left(a_{n}-\gamma_{\mathrm{th}}\tilde{a}\right)},XY\geq \frac{\gamma_{\mathrm{th}}\sigma_{D,n}^{2}}{P_{S}\phi \left(b_{n}-\gamma_{\mathrm{th}}\tilde{b}\right)}\right), \end{array}$$

where $\phi =\frac{2\alpha \eta }{1-\alpha }$, $\tilde{a}=\sum_{i=n+1}^{N}a_{i}$ and $\tilde{b}=\sum_{i=n+1}^{N}b_{i}$. The $\overset{\Delta }{=}$ means that conditions, the $a_{n}>\gamma_{\mathrm{th}}\tilde{a}$ and $b_{n}>\gamma_{\mathrm{th}}\tilde{b}$, need to be satisfied. If not, $a_{n}\leq \gamma_{\mathrm{th}}\tilde{a}$ or $b_{n}\leq \gamma_{\mathrm{th}}\tilde{b}$, the outage always happens because X,Y∈(0,∞) is considered only. Hence, the power allocated for the $D_{n}$ is more than the power of the others.

Based on the theory of joint probability of two random variables [41], the Equation (29) can be represented as

(30) $$\begin{array}{cc} {\mathrm{OP}}_{D_{n}} & =1-\int_{u}^{\infty }\left[1-F_{Y}\left(\frac{v}{x}\right)\right]f_{X}\left(x\right)dx\\ & =1-\underset{I_{1}}{\underset{︸}{\int_{u}^{\infty }f_{X}\left(x\right)dx}}+\underset{I_{2}}{\underset{︸}{\int_{u}^{\infty }F_{Y}\left(\frac{v}{x}\right)f_{X}\left(x\right)dx}}, \end{array}$$

where $u=\frac{\gamma_{\mathrm{th}}\sigma_{R}^{2}}{P_{S}\left(a_{n}-\gamma_{\mathrm{th}}\tilde{a}\right)}$ and $v=\frac{\gamma_{\mathrm{th}}\sigma_{D,n}^{2}}{P_{S}\phi \left(b_{n}-\gamma_{\mathrm{th}}\tilde{b}\right)}$.

Substituting (23) into (30), we obtain $I_{1}$ as

(31) $$\begin{array}{c} I_{1}=\frac{1}{\Gamma \left(K\right){\left[\left(1-\rho \right)\Omega_{\mathrm{SR}}\right]}^{K}}\int_{u}^{\infty }x^{K-1}\exp\left(-\frac{x}{\left(1-\rho \right)\Omega_{\mathrm{SR}}}\right)dx. \end{array}$$

Thank to the help of ([32], Equation (3.351.2)), we have

(32) $$\begin{array}{c} I_{1}=\frac{1}{\Gamma (K)}\Gamma \left(K,\frac{\gamma_{\mathrm{th}}\sigma_{R}^{2}}{P_{S}\left(a_{n}-\gamma_{\mathrm{th}}\tilde{a}\right)\left(1-\rho \right)\Omega_{\mathrm{SR}}}\right). \end{array}$$

Substituting (23) and (28) into (30), we obtain $I_{2}$ as in (33).

(33) I2=N!(N−n)!(n−1)!∑j=0N−n(−1)jn+jN−nj∑ℓ=0n+j(−1)ℓn+jℓ1Γ(K)[(1−ρ)ΩSR]K×∫u∞xK−1exp−ℓvx(1−ρ)ΩRDjexp−x(1−ρ)ΩSRdx.

To calculate exactly $I_{2}$, we can use expansion in series for exponential function as in [42], i.e., $\exp\left(-\frac{a}{x}\right)=\sum_{t=0}^{\infty }\frac{{\left(-1\right)}^{t}}{t!}{\left(\frac{a}{x}\right)}^{t}$. Thus, $I_{2}$ is rewritten by

(34) I2=N!(N−n)!(n−1)!∑j=0N−n(−1)jn+jN−nj∑ℓ=0n+j(−1)ℓn+jℓ1Γ(K)[(1−ρ)ΩSR]K×∑t=0∞(−1)tt!ℓv(1−ρ)ΩRDjt∫u∞xqexp−x(1−ρ)ΩSRdx︸Δ1,whereq=K−1−t.

To calculate $\Delta_{1}$ in (34), we consider two cases of power index, i.e., q≥0 and q<0. Using ([32], Equation (3.351.2)) for the case of q≥0 and exponential integral function for the case of q<0. The exponential integral function have been defined by Schloemilch as in ([43], [5.1.4]).

(35) $$\begin{array}{c} E_{n}\left(z\right)=\int_{1}^{\infty }\frac{e^{-zt}}{t^{n}}dt. \end{array}$$

Finally, we have $\Delta_{1}$ as in (36) and (37).

(36) $$\begin{array}{cc} & \Delta_{1}={\left(\frac{1}{\left(1-\rho \right)\Omega_{\mathrm{SR}}}\right)}^{-q-1}\Gamma \left(q+1,\frac{u}{\left(1-\rho \right)\Omega_{\mathrm{SR}}}\right),\mathrm{if}\;q\geq 0. \end{array}$$

(37) $$\begin{array}{cc} & \Delta_{1}={\left(\frac{1}{u}\right)}^{q-1}E_{q}\left(\frac{u}{\left(1-\rho \right)\Omega_{\mathrm{SR}}}\right),\mathrm{if}\;q<0 \end{array}$$

In the case of the transmit power is high enough, i.e., $u=\frac{\gamma_{\mathrm{th}}\sigma_{R}^{2}}{P_{S}\left(a_{n}-\gamma_{\mathrm{th}}\tilde{a}\right)}\to 0$, we have $I_{1}=1$. Hence, the closed-form of ${\mathrm{OP}}_{D_{n}}$ can be simplified as follows.

(38) OPDn≈N!(N−n)!(n−1)!∑j=0N−n(−1)jn+jN−nj∑ℓ=0n+j(−1)ℓn+jℓ1Γ(K)[(1−ρ)ΩSR]K×∫0∞xK−1exp−ℓvx(1−ρ)ΩRDj−x(1−ρ)ΩSRdx.

Based on ([32], Equation (3.471.9)), an approximation of $I_{2}$ is evaluated as

(39) OPDn≈N!(N−n)!(n−1)!∑j=0N−n(−1)jn+jN−nj∑ℓ=0n+j(−1)ℓn+jℓ1Γ(K)[(1−ρ)ΩSR]K×2ℓvΩSRΩRDjK2KK4ℓv(1−ρ)2ΩSRΩRDj,

where $K_{K}$ expresses the modified Bessel function of the second kind of order *K*.

On the other hand, in the case of imperfect SIC, (21) can be rewritten as

(40) $$\begin{array}{c} {\mathrm{OP}}_{D_{n}}^{\mathrm{ipSIC}}\overset{\Delta }{=}1-\mathrm{Pr}\left(\begin{array}{c} X\geq \frac{\gamma_{\mathrm{th}}\sigma_{R}^{2}}{P_{S}\left[a_{n}-\gamma_{\mathrm{th}}\left(\beta_{1}+\beta_{2}\right)\right]},\\ XY\geq \frac{\gamma_{\mathrm{th}}\sigma_{D,n}^{2}}{P_{S}\phi \left[b_{n}-\gamma_{\mathrm{th}}\left(\psi_{1}+\psi_{2}\right)\right]} \end{array}\right), \end{array}$$

where $\beta_{1}=\sum_{i=n+1}^{N}a_{i}$, $\beta_{2}=\xi_{1}\sum_{k=1}^{n-1}a_{k}$ and $\psi_{1}=\sum_{i=n+1}^{N}b_{i}$, $\psi_{2}=\xi_{2}\sum_{k=1}^{n-1}b_{k}$.

Similarly to the case of perfect SIC, based on the theory of joint probability of two random variable function, we have the OP under imperfect SIC as

(41) $$\begin{array}{cc} {\mathrm{OP}}_{D_{n}}^{\mathrm{ipSIC}} & =1-\int_{\mu }^{\infty }\left[1-F_{Y}\left(\frac{\lambda }{x}\right)\right]f_{X}\left(x\right)dx\\ & =1-\underset{J_{1}}{\underset{︸}{\int_{\mu }^{\infty }f_{X}\left(x\right)dx}}+\underset{J_{2}}{\underset{︸}{\int_{\mu }^{\infty }F_{Y}\left(\frac{\lambda }{x}\right)f_{X}\left(x\right)dx}}, \end{array}$$

where $\mu =\frac{\gamma_{\mathrm{th}}\sigma_{R}^{2}}{P_{S}\left[a_{n}-\gamma_{\mathrm{th}}\left(\beta_{1}+\beta_{2}\right)\right]}$ and $\lambda =\frac{\gamma_{\mathrm{th}}\sigma_{D,n}^{2}}{P_{S}\phi \left[b_{n}-\gamma_{\mathrm{th}}\left(\psi_{1}+\psi_{2}\right)\right]}$.

Similar to the case of perfect SIC and based on CDF and PDF given in (22) and (23), the result of imperfect SIC is described as follows.

(42) $$\begin{array}{c} J_{1}=\frac{1}{\Gamma (K)}\Gamma \left(K,\frac{\gamma_{\mathrm{th}}\sigma_{R}^{2}}{P_{S}\left[a_{n}-\gamma_{\mathrm{th}}\left(\beta_{1}+\beta_{2}\right)\right]\left(1-\rho \right)\Omega_{\mathrm{SR}}}\right). \end{array}$$

Besides, $J_{2}$ can be obtained as follows.

(43) J2=N!(N−n)!(n−1)!∑j=0N−n(−1)jn+jN−nj∑ℓ=0n+j(−1)ℓn+jℓ1Γ(K)[(1−ρ)ΩSR]K×∑t=0∞(−1)tt!ℓλ(1−ρ)ΩRDjt∫μ∞xqexp−x(1−ρ)ΩSRdx︸Δ2,whereq=K−1−t.

By replacing the inner parameters for the corresponding variables, we have $\Delta_{2}$ as in (44) and (45).

(44) $$\begin{array}{cc} & \Delta_{2}={\left(\frac{1}{\left(1-\rho \right)\Omega_{\mathrm{SR}}}\right)}^{-q-1}\Gamma \left(q+1,\frac{\gamma_{\mathrm{th}}\sigma_{R}^{2}}{\left(1-\rho \right)\Omega_{\mathrm{SR}}P_{S}\left[a_{n}-\gamma_{\mathrm{th}}\left(\beta_{1}+\beta_{2}\right)\right]}\right),\mathrm{if}\;q\geq 0. \end{array}$$

(45) $$\begin{array}{cc} & \Delta_{2}={\left(\frac{P_{S}\left[a_{n}-\gamma_{\mathrm{th}}\left(\beta_{1}+\beta_{2}\right)\right]}{\gamma_{\mathrm{th}}\sigma_{R}^{2}}\right)}^{q-1}E_{q}\left(\frac{\gamma_{\mathrm{th}}\sigma_{R}^{2}}{\left(1-\rho \right)\Omega_{\mathrm{SR}}P_{S}\left[a_{n}-\gamma_{\mathrm{th}}\left(\beta_{1}+\beta_{2}\right)\right]}\right),\mathrm{if}\;q<0 \end{array}$$

### 3.2. Ergodic Rate

**Figure 4:**
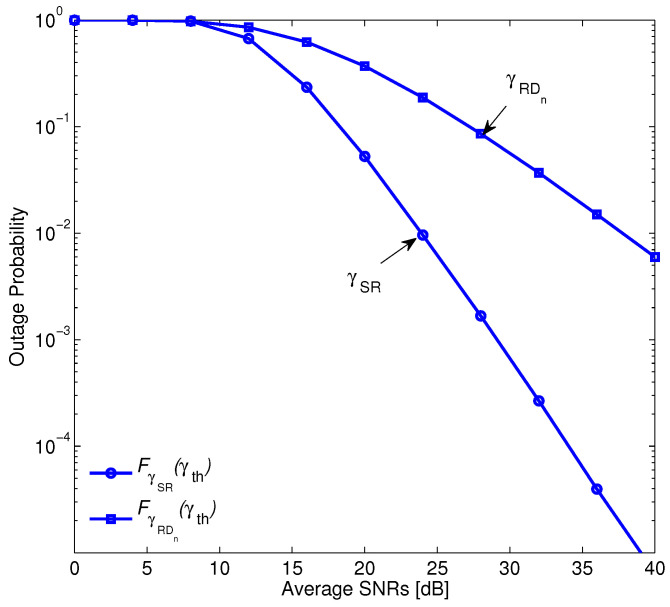
The comparison between CDF of $\gamma_{\mathrm{SR}}$ and CDF of $\gamma_{\mathrm{RD}}$.

Before investigating the ergodic rate of the system, we examine the result that had been shown in [44], i.e., the transmission power of the relay is often smaller than the transmission power of the BS because the relay node is supplied by harvesting energy. In Figure 4, the probabilities of $\gamma_{\mathrm{SR}}$ and $\gamma_{\mathrm{RD}}$ are compared. It is clear that $\gamma_{\mathrm{RD}}$ is always less than $\gamma_{\mathrm{SR}}$. Therefore, the probability that $\gamma_{{\mathrm{RD}}_{n}}<\gamma_{\mathrm{th}}$ is always higher than the probability that $\gamma_{\mathrm{SR}}<\gamma_{\mathrm{th}}$. This ensures that $\min\left(\gamma_{\mathrm{SR}},\gamma_{{\mathrm{RD}}_{n}}\right)=\gamma_{{\mathrm{RD}}_{n}}$ and it can be considered to be a crucial factor that limits the performance of such systems.

Based on the above analysis and Claude Shannon theory, the instantaneous data rate of (m,n)th user is given by

(46) $$\begin{array}{cc} R_{m,n}= & \frac{1-\alpha }{2}\log_{2}\left(1+\frac{P_{R}b_{n}{\left|g_{n}\right|}^{2}}{\tilde{b}P_{R}|g_{n}{|}^{2}+\psi_{2}P_{R}{\left|g_{n}\right|}^{2}+\sigma_{D,n}^{2}}\right)\\ = & \frac{1-\alpha }{2}\log_{2}\left(\frac{P_{R}{\left|g_{n}\right|}^{2}\left(\tilde{b}+\psi_{2}+b_{n}\right)+\sigma_{D,n}^{2}}{\tilde{b}P_{R}|g_{n}{|}^{2}+\psi_{2}P_{R}{\left|g_{n}\right|}^{2}+\sigma_{D,n}^{2}}\right). \end{array}$$

We can rewrite (46) as

(47) $$\begin{array}{cc} R_{m,n}= & \frac{1-\alpha }{2}\log_{2}\left(1+\phi \Psi |h_{m}w_{m}{|}^{2}{\left|g_{n}\right|}^{2}\left(\tilde{b}+\psi_{2}+b_{n}\right)\right)-\frac{1-\alpha }{2}\log_{2}\left(1+\phi \Psi |h_{m}w_{m}{|}^{2}{\left|g_{n}\right|}^{2}\left(\tilde{b}+\psi_{2}\right)\right), \end{array}$$

where $\Psi =\frac{P_{S}}{\sigma_{D_{n}}^{2}}$ is the received signal to noise ratio.

To simplify, we let $\Gamma_{1}=\phi \Psi |h_{m}w_{m}{|}^{2}{\left|g_{n}\right|}^{2}\left(\tilde{b}+\psi_{2}+b_{n}\right)$ and $\Gamma_{2}=\phi \Psi |h_{m}w_{m}{|}^{2}{\left|g_{n}\right|}^{2}\left(\tilde{b}+\psi_{2}\right)$. Thus, we have CDFs of $\Gamma_{1}$ and $\Gamma_{2}$ given as

(48) FΓ1(γ1)=1−∑n=1NNn2(−1)n−1Γ(K)[(1−ρ)ΩSR]Knγ1ΩSRΩRDjϕΨBK2KK4nγ1(1−ρ)2ΩSRϕΨBΩRDj,

and

(49) FΓ2(γ2)=1−∑n=1NNn2(−1)n−1Γ(K)[(1−ρ)ΩSR]Knγ2ΩSRΩRDjϕΨCK2KK4nγ2(1−ρ)2ΩSRϕΨCΩRDj,

where $B=(\tilde{b}+b_{n}+\psi_{2})$, $C=(\tilde{b}+\psi_{2})$. $F_{\Gamma_{1}}\left(\gamma \right)$ and $F_{\Gamma_{2}}\left(\gamma \right)$ are solved in Appendix A.

Based on (47), the ergodic rate can be calculated as

(50) $$\begin{array}{cc} E[R_{m,n}]= & \underset{C_{1}}{\underset{︸}{\frac{1-\alpha }{2}E\left[\log_{2}\left(1+\Gamma_{1}\right)\right]}}-\underset{C_{2}}{\underset{︸}{\frac{1-\alpha }{2}E\left[\log_{2}\left(1+\Gamma_{2}\right)\right]}}. \end{array}$$

Expression (50) follows the strictly monotonically increasing property of the logarithm function for non-negative real numbers.

(51) $$\begin{array}{cc} C_{1} & =\frac{1-\alpha }{2\ln2}\int_{0}^{\infty }\log_{2}\left(1+\Gamma_{1}\right)f_{\Gamma_{1}}\left(\gamma_{1}\right)d\gamma_{1}. \end{array}$$

Based on the properties of the expected value of a random variable, using the integration-by-parts method, we have

(52) $$\begin{array}{cc} C_{1} & =\frac{1-\alpha }{2\ln2}\int_{0}^{b}\log_{2}\left(1+\Gamma_{1}\right)d\left[F_{\Gamma_{1}}\left(\gamma_{1}\right)-1\right]\\ & =\frac{1-\alpha }{2\ln2}\left(\log_{2}\left(1+\Gamma_{1}\right)\left[F_{\Gamma_{1}}\left(b\right)-1\right]\right)-\frac{1-\alpha }{2\ln2}\int_{0}^{b}\frac{F_{\Gamma_{1}}\left(\gamma_{1}\right)-1}{1+\gamma_{1}}d\gamma_{1}. \end{array}$$

$F_{\Gamma_{1}}\left(\gamma_{1}\right)$ is CDF of $\Gamma_{1}$, thus when *b* is a finite value, it is seen that $\log_{2}\left(1+\Gamma_{1}\right)\left[F_{\Gamma_{1}}\left(b\right)-1\right]=0$. Thus, $C_{1}$ is given as

(53) $$\begin{array}{cc} C_{1} & =\frac{1-\alpha }{2\ln2}\int_{0}^{\infty }\frac{1-F_{\Gamma_{1}}\left(\gamma_{1}\right)}{1+\gamma_{1}}d\gamma_{1}. \end{array}$$

Similar to the working step in (51), we can rewrite the second part as the following equation.

(54) $$\begin{array}{cc} C_{2} & =\frac{1-\alpha }{2\ln2}\int_{0}^{\infty }\log_{2}\left(1+\Gamma_{2}\right)f_{\Gamma_{2}}\left(\gamma_{2}\right)d\gamma_{2}\\ & =\frac{1-\alpha }{2\ln2}\int_{0}^{\infty }\frac{1-F_{\Gamma_{2}}\left(\gamma_{1}\right)}{1+\gamma_{2}}d\gamma_{2}, \end{array}$$

where the $F_{\Gamma_{1}}\left(\gamma \right)$ and $F_{\Gamma_{2}}\left(\gamma \right)$ are given in (48) and (49).

With the help of ([32], Equation (7.811.5)) and ([32], Equation (9.343)), after some manipulations, we have $C_{1}$ and $C_{2}$ as

(55) C1=1−α2ln2∑n=1NNn(−1)n−1Γ(K)[(1−ρ)ΩSR]KnΩSRΩRDjϕΨBK2G1,33,1n(1−ρ)2ΩSRϕΨBΩRDj−K2,−K2,K2−K2,

and

(56) C2=1−α2ln2∑n=1NNn(−1)n−1Γ(K)[(1−ρ)ΩSR]KnΩSRΩRDjϕΨCK2G1,33,1n(1−ρ)2ΩSRϕΨCΩRDj−K2,−K2,K2−K2.

The proof is shown in Appendix A.

## 4. Numerical Results

In this section, the performance of the proposed SWIPT-NOMA system in terms of the outage probability and ergodic rate is numerically analyzed. The simulation parameters are set as follows. The Monte Carlo simulations is run with $2\times 10^{14}$ trials. The variance of noise component at all receiving nodes is assumed to be constant, $\sigma^{2}=1$. The average channel gain $E\{|h_{m}{|}^{2}\}=\Omega_{{\mathrm{SR}}_{m}}=E\{|g_{1}{|}^{2}\}=\Omega_{R_{m}D_{1}}=1$, $E\{|g_{2}{|}^{2}\}=\Omega_{R_{m}D_{2}}=2$ and $E\{|g_{3}{|}^{2}\}=\Omega_{R_{m}D_{3}}=4$.

The threshold data rates of $D_{n}$: $r_{1}=r_{2}=r_{3}=1\left[b/s/\mathrm{Hz}\right]$. The energy conversion efficiency coefficient of R: η=0.85. The number of relay nodes: M=3 and each cluster has three users severed instantaneously by each relay node. The power allocation coefficient of the *n*th user: $a_{n}=\left(N-n+1\right)/\mu$, where μ is chosen such that $\sum_{n=1}^{N}\sqrt{a_{n}P_{S}}=1$. To simplify the system design and settings, we assume that the power allocation coefficients at the BS and the relay nodes are the same.

**Figure 5:**
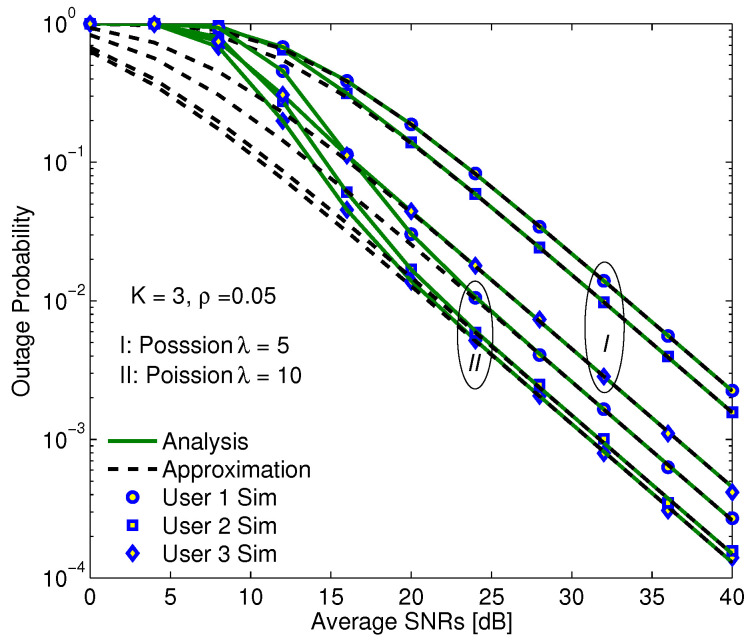
The OP versus SNRs of users with parameters as α = 0.3, η = 0.85, $a_{1}\;=\;0.6,\;a_{2}\;=\;0.3,\;a_{3}\;=\;0.1$.

Firstly, users are grouped randomly into clusters according to Poisson distribution in two cases of the λ parameter values, λ can be considered to be an expected value of the number of such events, in which an event is that a user appears in a cluster. Only three users with the best channel conditions are supported by the relay node at the same time, and the OPs of every user in both cases of λ are illustrated in Figure 5. As can be seen from this figure, if the transmission power is high enough, the OPs evaluated by exact analysis and approximation are the same, demonstrating that the proposed methods to calculate the OP are significantly reasonable and actually accurate. Moreover, while the transmission power is assigned for signal of user 1 with the highest value and user 3 with the lowest value, the OP of user 3 is the lowest, in other words, user 3 has the best quality. The reason is that the total interference affected to user 1 consists of signals of both user 2 and user 3, and its own noise, while the total interference influenced on user 3 contains its own noise only owing to the perfect SIC. Furthermore, the system performance is improved if the λ parameter increases. The reason is explained as when the λ rises, the number of users appears in a cluster goes up, resulting in the selection of three users with the best channel conditions is more efficient.

**Figure 6:**
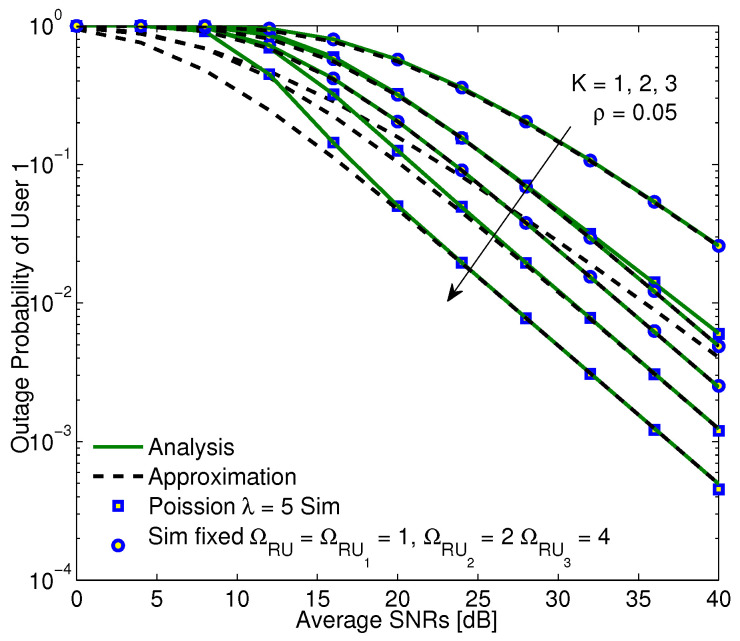
The OP versus SINRs with different numbers of receiving antennas and α = 0.3, η = 0.85, $a_{1}\;=\;0.6,\;a_{2}\;=\;0.3,\;a_{3}\;=\;0.1$.

In Figure 6, the proposed system is investigated in such scenario that users are fixed and randomly grouped according to Poison rule with the average value of the λ parameter. It is clear that when the number of transmitting antennas of the BS increases, the system performance is enhanced. There are two reasons, i.e., the diversity gain of the link between BS-R is improved and the harvested energy at the relay node rises or transmission power of the relay node rises and then the messages are sent from the relay node more dependably. In addition, the system performance in case of random user distribution may be better than that in case of fixed user distribution. The reason is that if users are fixed, the channel gains are likely to be fixed and then the selection of three users with best channel condition is less effective than that in case of random distribution. Finally, the results in exact analysis and approximation are absolutely the same at high enough SNR region, once again, confirming the accuracy of the proposed methods.

**Figure 7:**
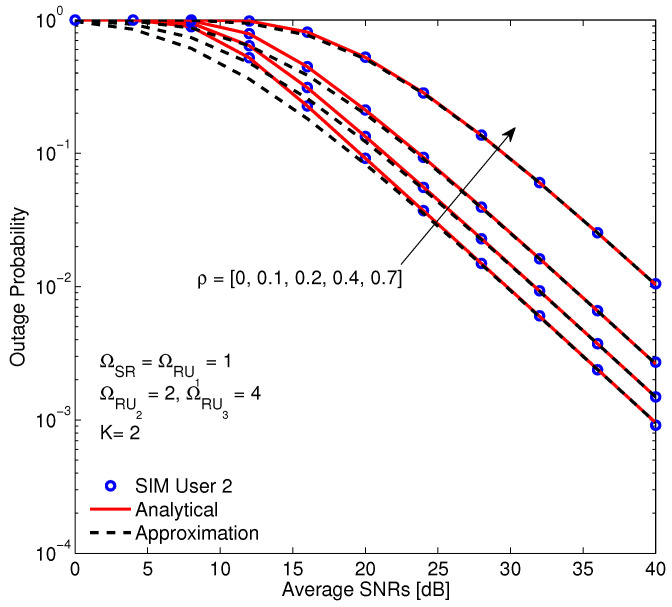
The OP versus average SNR with different correlation coefficients and α = 0.3, η = 0.85, $a_{1}\;=\;0.6,a_{2}\;=\;0.3,a_{3}\;=\;0.1$.

The effect of channel estimation error on the OP of the system is illustrated in Figure 7. The curves show that increasing ρ, i.e., increasing estimation error, results in a reduction of the OP performance. This result demonstrates that the channel estimation error influences significantly on the system performance. In fact, the instantaneous CSI not only affects to coding gain but also causes leakage beam also. Finally, the Monte Carlo simulation guarantees the correctness of the exact and approximation analytical results.

**Figure 8:**
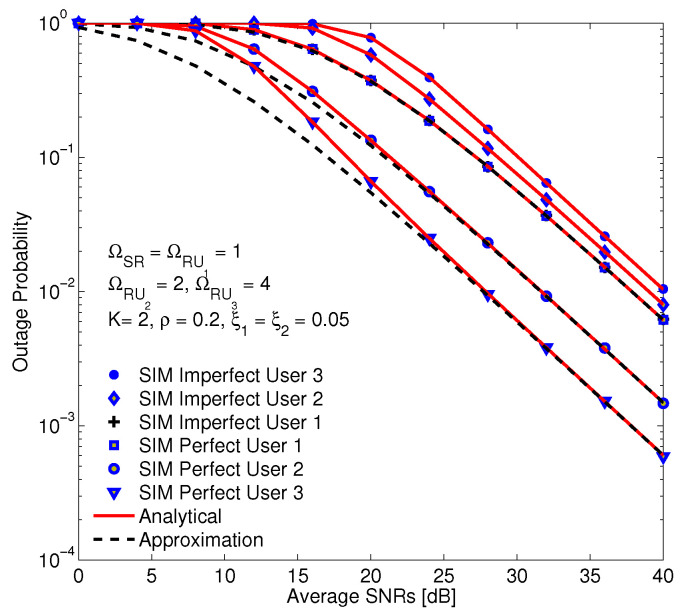
The OP of users in term of imperfect and perfect SIC with residual coefficients of SIC are $\xi_{1}\;=\;\xi_{2}\;=\;0.05$, and α = 0.3, η = 0.85, $a_{1}\;=\;0.6,a_{2}\;=\;0.3,a_{3}\;=\;0.1$.

In Figure 8, the OP of users versus SNR in two cases of perfect and imperfect SICs are represented. In this figure, the OP of imperfect SIC is higher than that of perfect SIC and the OP of user 1 are the same in two cases. In fact, user 1 does not employ the SIC structure, it decodes its own signal by considering the other users’ signals as interference. However, the residual power of user 1 impacts to the decoding process of the other users. Different from the case of perfect SIC, the quality of user 3 is least of all among users in the scenario of imperfect SIC. It is because the residual power, that plays as interference, at user 3, it is the largest. The provided simulation results confirm the accuracy of the proposed analytical method, and the approximation is the same with simulation in the medium and high SINR regions.

**Figure 9:**
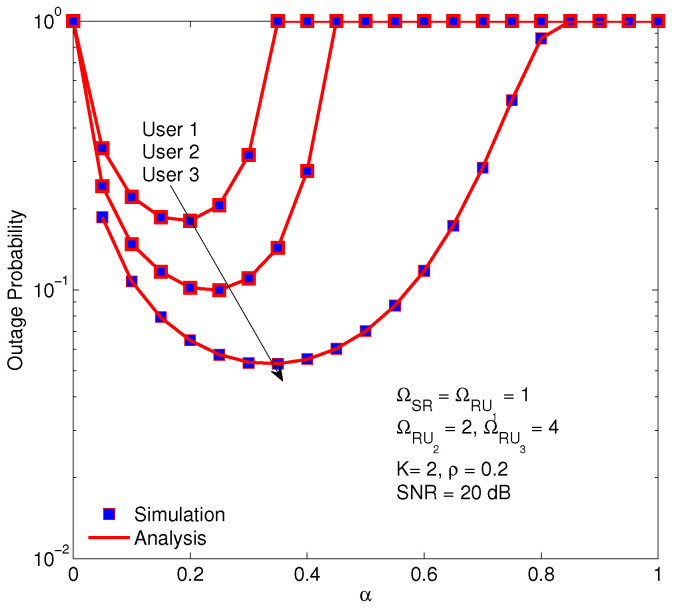
The optimal time fraction of EH corresponding to minimum OP of users with η = 0.85, $a_{1}\;=\;0.6,a_{2}\;=\;0.3,a_{3}\;=\;0.1$.

Figure 9 demonstrates the impact of the time fraction of EH, 0<α<1, on the OP of users. In fact, the optimal α is a sophisticated function of the channel and system parameters. The smaller α results in the smaller energy for relay nodes, however, the larger α brings the smaller transmission power for communication of the BS. Therefore, if the α increases from 0 to 1, the OP reduces to an optimal value and then increases again. As a result, the optimal α corresponding to the smallest OP is determined by this plot. In the figure, the minimum values of OP for every user are different although the setting parameters are the same. Moreover, the optimal time duration for EH of user 3 is the largest. The reason is that the power allocation for user 3 is the smallest, consequently, it needs longer time duration for EH to ensure the transmission power of the relay node to forward signals.

**Figure 10:**
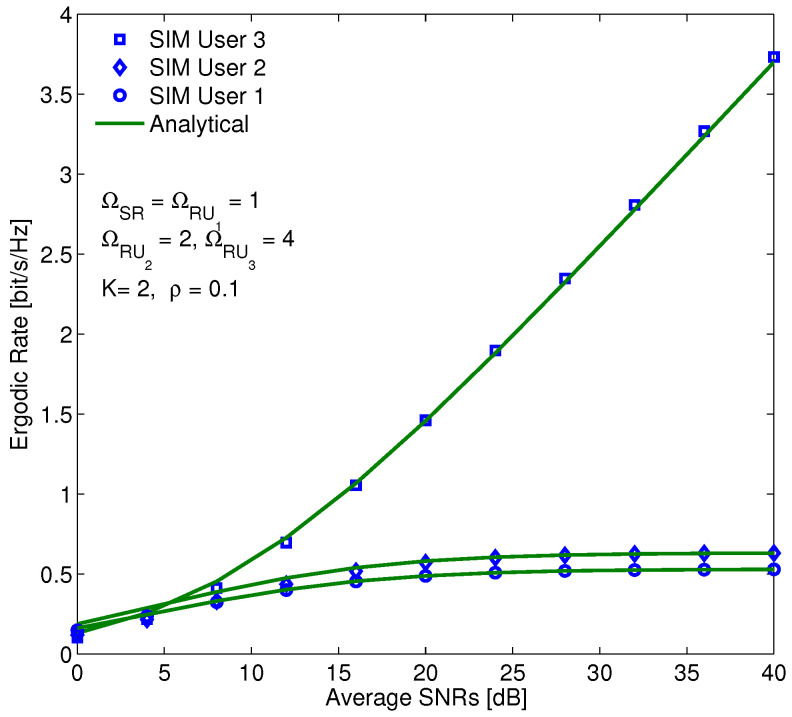
Compare the ER of users in each cluster for the perfect SIC with η = 0.85, $a_{1}\;=\;0.6,$ $a_{2}\;=\;0.3,$ $a_{3}\;=\;0.1$.

Figure 10 demonstrates the ER of users versus SINR in the case of perfect SIC. In this figure, the ER can be considered to be a function of the mean SINR, based on the derived results in (55) and (56). Figure 10 shows that the ERs of users 1 and 2 increase negligibly in the low SINR region and remain stable in the high SINR region; however, that of user 3 increases exponentially. There is a trade-off between the complexity and the performance, i.e., user 1 directly detected its own signal, whereas the users 2 and 3 have the first-order and second-order of the SIC, respectively.

**Figure 11:**
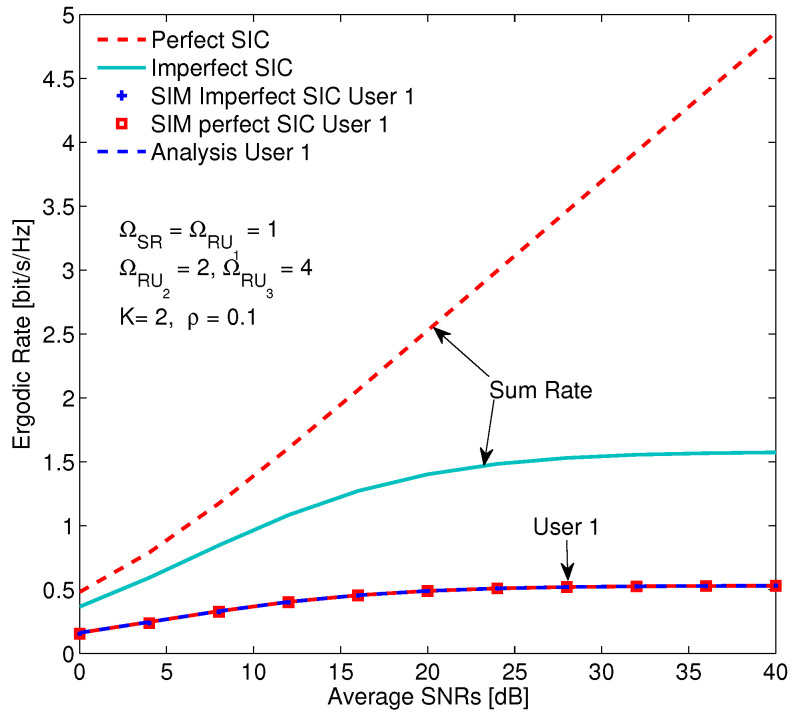
Sum ER of the system versus SINR in two cases of perfect and imperfect SICs with $\xi_{1}\;=\;\xi_{2}\;=\;0.06$.

The sum ER of the system is demonstrated in Figure 11 for two cases, i.e., perfect and imperfect SICs. Firstly, the simulation results are the same with analytical results. Secondly, the sum ER in the case of perfect SIC is higher than that in the case of imperfect SIC, especially the gap of them increases when the SINR increases. The reason can be explained as if SINR increases by rising the transmission power, the interference due to imperfect SIC also increases, consequently, the sum ER with imperfect SIC goes up less significantly than that with perfect SIC. Finally, the ERs of user 1 are the same in both cases because when user 1 decodes the signal, the SIC scheme is not used.

**Figure 12:**
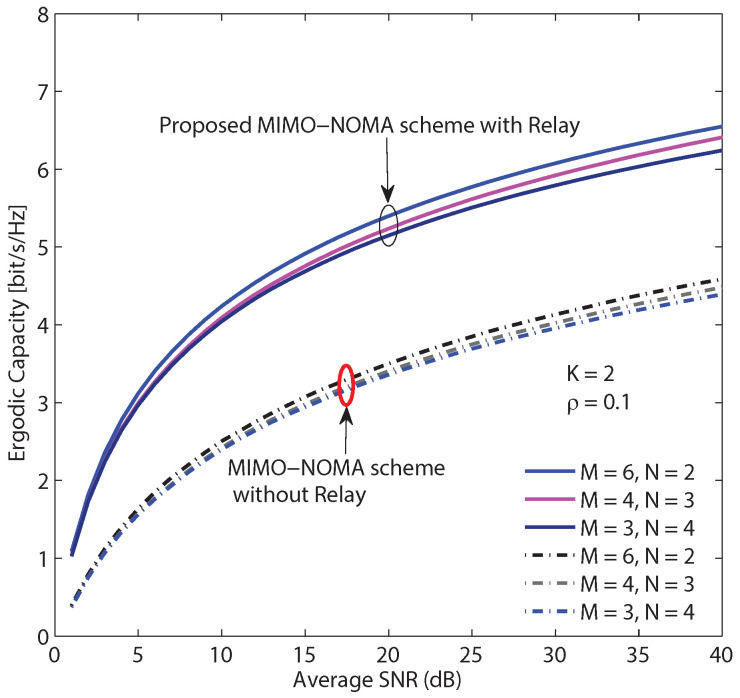
The comparison of 3 different user clustering modes with 12 users, i.e., (M = 6, N = 2), (M = 4, N = 3) and (M = 3, N = 4), and $\xi_{1}\;=\;\xi_{2}\;=\;0.06$.

Figure 12 demonstrates the sum ER of the system versus SNR with the following settings. The number of users is 12, there are three typical user clustering modes, and the imperfect SIC coefficients are $\xi_{1}=\xi_{2}=0.06$. As shown in Figure 12, the first mode has the best performance due to the lowest cross user interference. In contrast, the third mode achieves the worst performance because when the number of users in the cluster increases, the cross user interference rises. It is worth noting that the number of users and the number of clusters determine the number of beams and the order of SIC. As a result, we can dynamically select the user clustering mode according to system parameters and channel conditions for balancing the overall performance and the computational complexity. In addition, the proposed multiple relay aided MIMO-NOMA scheme and a MIMO-NOMA scheme without relay are compared in Figure 12. It is obvious that the proposed scheme has much better performance than the scheme without relay. In fact, the scheme without relay has lower channel gains than the proposed scheme, i.e., $\Omega_{\mathrm{BS}-D_{1}}=0.5,\Omega_{\mathrm{BS}-D_{2}}=1$ and $\Omega_{\mathrm{BS}-D_{3}}=2$, because of the longer distance from point to point (BS, R or D). Moreover, in the proposed scheme, the inter-relay interference can be effectively canceled.

## 5. Conclusions

In this paper, a combination design of NOMA, SWIPT, and beamforming is proposed for the downlink of multi-user relaying systems. To analyze and evaluate the system performance, the outage probability and ergodic capacity are derived based on analytics and statistics. The proposed system is investigated obviously in two cases of the perfect and imperfect SIC structures, and the effect of the imperfect CSI is also taken into consideration. Moreover, an optimal time fraction of energy harvesting is found out to minimize the outage probability and the reasonable user clustering mode is discussed. The analytical and simulation results demonstrate the accuracy of the proposed analysis method. In the future, the authors will investigate the system with a full-duplex model to improve the spectrum efficiency and apply the power splitting scheme to the SWIPT with the finite block-length.

## Figures and Tables

**Table 1:** The notations.

Notation	Description
Pr	Probability
$F_{X}\left(x\right)$	Cumulative distribution function (CDF)
$f_{X}\left(x\right)$	Probability density function (PDF)
$\mathrm{CN}(\mu ,\sigma^{2})$	A circularly symmetric complex Gaussian RV *x* with mean μ and variance $\sigma^{2}$
$E\left\{\cdot \right\}$	The statistical expectation operator
Γ(·)	Gamma function [32]
$K_{n}\left(\cdot \right)$	The second kind of Bessel function, order *n* [32]
$E_{n}\left(z\right)$	Exponential integral function *n* [32]
$G_{pq}^{mn}\left(x{|}_{b_{s}}^{a_{r}}\right)$	Meijer’s G-Function [9.3] [32]
*M*	Number of relay nodes
*N*	Number of users in each cluster
$N_{t},N_{r}$	Transmission antennas of BS and reception antennas of relay
$\rho_{A}$	Correlation coefficient
α	Time switching ratio
η	Conversion efficiency
*r*	Data rate threshold

## Abbreviations

The following abbreviations are used in this manuscript:

AF	amplify-and-forward
AWGN	Additive white Gaussian noise
BS	Base station
CDF	Cumulative distribution function.
CSI	Channel state information
DF	Decode-and-forward
EH	Energy harvesting
ER	Ergodic rate
IoT	Internet of Things
i.i.d	Independent and identically distributed
MIMO	Multiple input multiple output
MMSE	Minimum mean squared error
NOMA	Non-orthogonal multiple access
MU	Multi-user
OP	Outage probability
PDF	Probability density function
RF	Radio Frequency
SCI	Successive interference cancellation
SINR	Signal to interference plus noise ratio
SNR	Signal to noise ratio
SWIPT	Simultaneous wireless information and power transfer
TS	Time switching
ZFBF	Zero-force beamforming

## Appendix A

To obtain the closed-form expressions of ER, we begin with the joint probability of two random variables as follows.

In previous sections, let $\Gamma_{1}=\phi \Psi |h_{m}w_{m}{|}^{2}{\left|g_{n}\right|}^{2}\left(\tilde{b}+\psi_{2}+b_{n}\right)$ and $\Gamma_{2}=\phi \Psi |h_{m}w_{m}{|}^{2}{\left|g_{n}\right|}^{2}\left(\tilde{b}+\psi_{2}\right)$, we have

(A1) $$\begin{array}{cc} F_{\Gamma_{1}}\left(\gamma_{1}\right) & =\mathrm{Pr}\left(XY\leq \frac{\gamma_{1}}{\phi \Psi (\tilde{b}+b_{n}+\psi_{2})}\right)\\ & =\int_{0}^{\infty }\mathrm{Pr}\left(Y\leq \frac{\gamma_{1}}{x\phi \Psi (\tilde{b}+b_{n}+\psi_{2})}\right)f_{X}\left(x\right)dx. \end{array}$$

Note that $F_{|g_{n}{|}^{2}}\left(\infty \right)=1$ and we let y→∞. Hence, from (27), it is easy to have N!(N−n)!(n−1)!∑j=0N−n(−1)jn+jN−nj=1. Thus, we can rewrite (27) as

(A2) F|gn|2(y)=1−∑n=1NNn(−1)n−1exp−ny(1−ρ)ΩRDj.

Substituting (26) and (A2) into (A1), we have

(A3) FΓ1(γ1)=1−∑n=1NNn(−1)n−1Γ(K)[(1−ρ)ΩSR]KΦ1(γ1),

where

(A4) $$\begin{array}{c} \Phi_{1}\left(\gamma_{1}\right)=\int_{0}^{\infty }x^{K-1}\exp\left(-\frac{n\gamma_{1}}{x\left(1-\rho \right)\Omega_{{\mathrm{RD}}_{j}}\phi \Psi B}-\frac{x}{\left(1-\rho \right)\Omega_{\mathrm{SR}}}\right)dx, \end{array}$$

with $B=(\tilde{b}+b_{n}+\psi_{2})$.

Using ([32], Equation (3.471.9)), $\Phi_{1}\left(\gamma_{1}\right)$ is obtained as

(A5) $$\begin{array}{c} \Phi_{1}\left(\gamma_{1}\right)={\left(\frac{n\gamma_{1}\Omega_{\mathrm{SR}}}{\Omega_{{\mathrm{RD}}_{j}}\phi \Psi B}\right)}^{\frac{K}{2}}K_{K}\left(\sqrt{\frac{4n\gamma_{1}}{{\left(1-\rho \right)}^{2}\Omega_{\mathrm{SR}}\phi \Psi B\Omega_{{\mathrm{RD}}_{j}}}}\right). \end{array}$$

Similar to above steps, the CDF of $\Gamma_{2}$ is evaluated as

(A6) $$\begin{array}{cc} F_{\Gamma_{2}}\left(\gamma_{2}\right)= & \mathrm{Pr}\left(XY\leq \frac{\gamma_{2}}{\phi \Psi (\tilde{b}+\psi_{2})}\right)\\ = & \int_{0}^{\infty }\mathrm{Pr}\left(Y\leq \frac{\gamma_{2}}{x\phi \Psi (\tilde{b}+\psi_{2})}\right)f_{X}\left(x\right)dx. \end{array}$$

(A7) FΓ2(γ2)=1−∑n=1NNn(−1)n−1Γ(K)[(1−ρ)ΩSR]KΦ2(γ2),

where

(A8) $$\begin{array}{c} \Phi_{2}\left(\gamma_{2}\right)={\left(\frac{n\gamma_{2}\Omega_{\mathrm{SR}}}{\Omega_{{\mathrm{RD}}_{j}}\phi \Psi C}\right)}^{\frac{K}{2}}K_{K}\left(\sqrt{\frac{4n\gamma_{2}}{{\left(1-\rho \right)}^{2}\Omega_{\mathrm{SR}}\phi \Psi C\Omega_{{\mathrm{RD}}_{j}}}}\right), \end{array}$$

with $C=(\tilde{b}+\psi_{2})$.

Substituting (A6) and (A7) into (53) and (54), respectively, we have

(A9) C1=1−α2ln2∑n=1NNn(−1)n−1Γ(K)[(1−ρ)ΩSR]KnΩSRΩRDjϕΨBK2∫0∞γ1K21+γ1KK4nγ1(1−ρ)2ΩSRϕΨBΩRDjdγ1,

with ([32], Equation (9.343)),

(A10) $$\begin{array}{c} K_{v}\left(x\right)x^{\mu }=2^{\mu -1}G_{02}^{20}\left({\left.\frac{1}{4}x^{2}\right|}_{\frac{1}{2}\mu +\frac{1}{2}v,\frac{1}{2}\mu -\frac{1}{2}v}\right). \end{array}$$

Thank to the help of the ([32], Equation (7.811.5)) and after some manipulations, the average rate is derived as in (55).

To find the average rate $C_{2}$, a similar procedure is performed, and proof is completed.
